# Complete genome sequence of the *Robinia pseudoacacia* L. symbiont *Mesorhizobium amorphae* CCNWGS0123

**DOI:** 10.1186/s40793-018-0321-3

**Published:** 2018-09-18

**Authors:** Xinye Wang, Yantao Luo, Dongying Liu, Jiamei Wang, Shi Wei, Liang Zhao

**Affiliations:** 1 Department of Liquor Making Engineering, Moutai College, 564500 Renhuai, People’s Republic of China; 2 State Key Laboratory of Crop Stress Biology for Arid Areas, College of Life Science, Northwest A&F University, 712100 Yangling, China

**Keywords:** Rhizobia, Symbiosis, Nodulation, Nitrogen

## Abstract

*Mesorhizobium amorphae* CCNWGS0123 was isolated in 2006, from effective nodules of *Robinia pseudoacacia* L. grown in lead-zinc mine tailing site, in Gansu Province, China. *M. amorphae* CCNWGS0123 is an aerobic, Gram-negative, non-spore-forming rod strain. This paper characterized *M. amorphae* CCNWGS0123 and presents its complete genome sequence information and genome annotation. The 7,374,589 bp long genome which encodes 7136 protein-coding genes and 63 RNA coding genes, contains one chromosome and four plasmids. Moreover, a chromosome with no gaps was assembled.

**Electronic supplementary material:**

The online version of this article (10.1186/s40793-018-0321-3) contains supplementary material, which is available to authorized users.

## Introduction

Soil microorganism - rhizobia (root nodule bacteria) could establish a symbiotic relationship with *Leguminosae* plants, forming a special organ - root nodule, the bacteroid in the root nodules converts atmospheric N_2_ into ammonium [1, 2]. The ammonium could help the host plants in surviving in N-limited environmental conditions [3]; in turn, host plants could provide the rhizobia with carbon and energy source for their growth and functions [4]. Establishment of this symbiosis requires successful infection in legume roots, and such infection is a multifaceted developmental process driven by the bacteria, but is ultimately under the control of the host [5]. This mutualistic association is highly specific such that each rhizobial species/strain interacts only with a specific group of legumes, and vice versa [6],this phenomenon is termed as symbiosis specificity. *Rhizobium leguminosarum* bv. *trifolii* WSM1325 could nodulate a diverse range of annual *Trifolium* (clover) species [7]. *Robinia pseudoacacia* L. are nodulated by *Mesorhizobium* and *Sinorhizobium* species which shared similar nodulation genes [8].

*Mesorhizobium amorphae* CCNWGS0123 was isolated from the root nodules of *R. pseudoacacia* L. grown in lead-zinc mine tailing site in Gansu Province, China [9]. The strain could promote the survival of its host plant in copper-, zinc- and chromium-contaminated environments [10]. The heavy metal tolerance and resistance mechanism of this strain has been investigated in previously studies [9, 11, 12].

In Chen’s study, they found that *M. amorphae* CCNWGS0123 nodulate with *R. pseudoacacia* L. [13]. The *M. amorphae* CCNWGS0123-*R. pseudoacacia* L. symbiosis system was selected to establish a rhizobium-legume symbiosis signal network. In order to provide some basis for the signal network establishment, the complete genome sequence and annotation of *M. amorphae* CCNWGS0123 genome were reported in this study.

## Organism information

### Classification and features

**Fig. 1:**
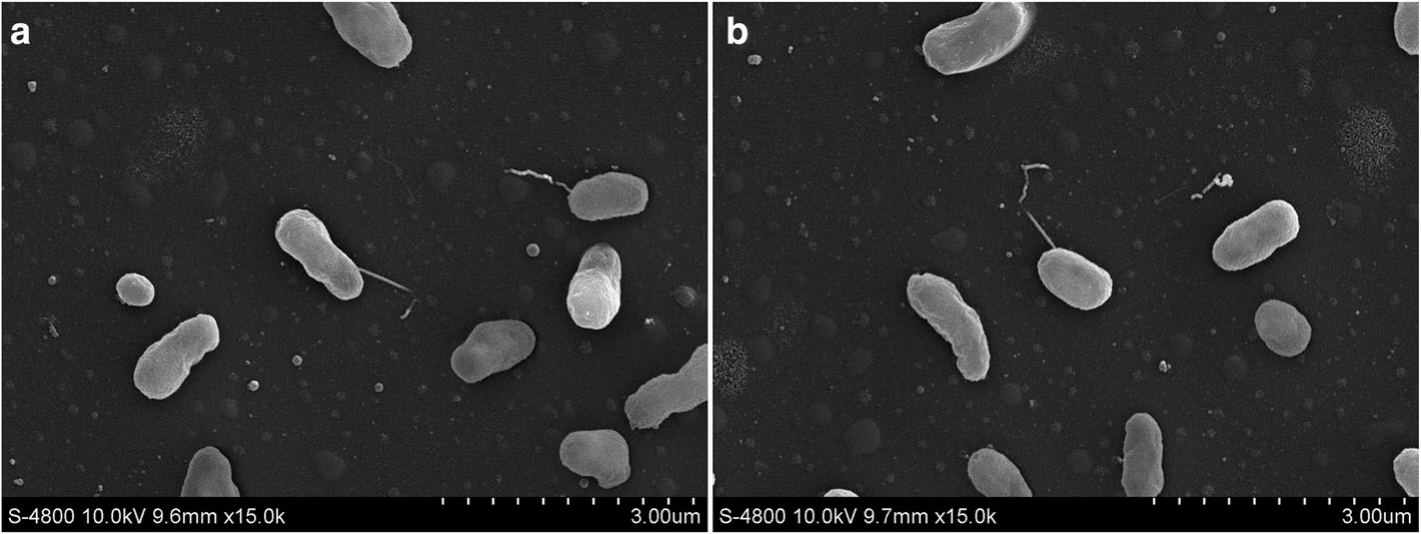
SEM (Scanning electron microscope) micrograph of *M. amorphae* CCNWGS0123 cells (**a**) and *M. amorphae* ACCC 19665^T^ (**b**)

*M. amorphae* CCNWGS0123 was isolated in 2006, from root nodules collected from *R. pseudoacacia* L. growing in lead-zinc mine tailing site in Gansu Province, China. *M. amorphae* CCNWGS0123 is a motile, non-spore forming, non-encapsulated, Gram-negative bacteria in the order *Rhizobiales* of the class *Alphaproteobacteria*. The rod-shaped bacterium is 0.41–0.65 μm wide and 0.47–1.68 μm long (Fig. 1a). *M. amorphae* CCNWGS0123 is nearly morphologically similar to *M. amorphae* ACCC 19665T (Fig. 1b). Colonies on solid media are circular, and translucent with a diameter of 1 mm growing for 7 days at 28 °C, the generation times range from 6 h to 13 h in YM broth as described by Wang in 1999 [14].

**Fig. 2:**
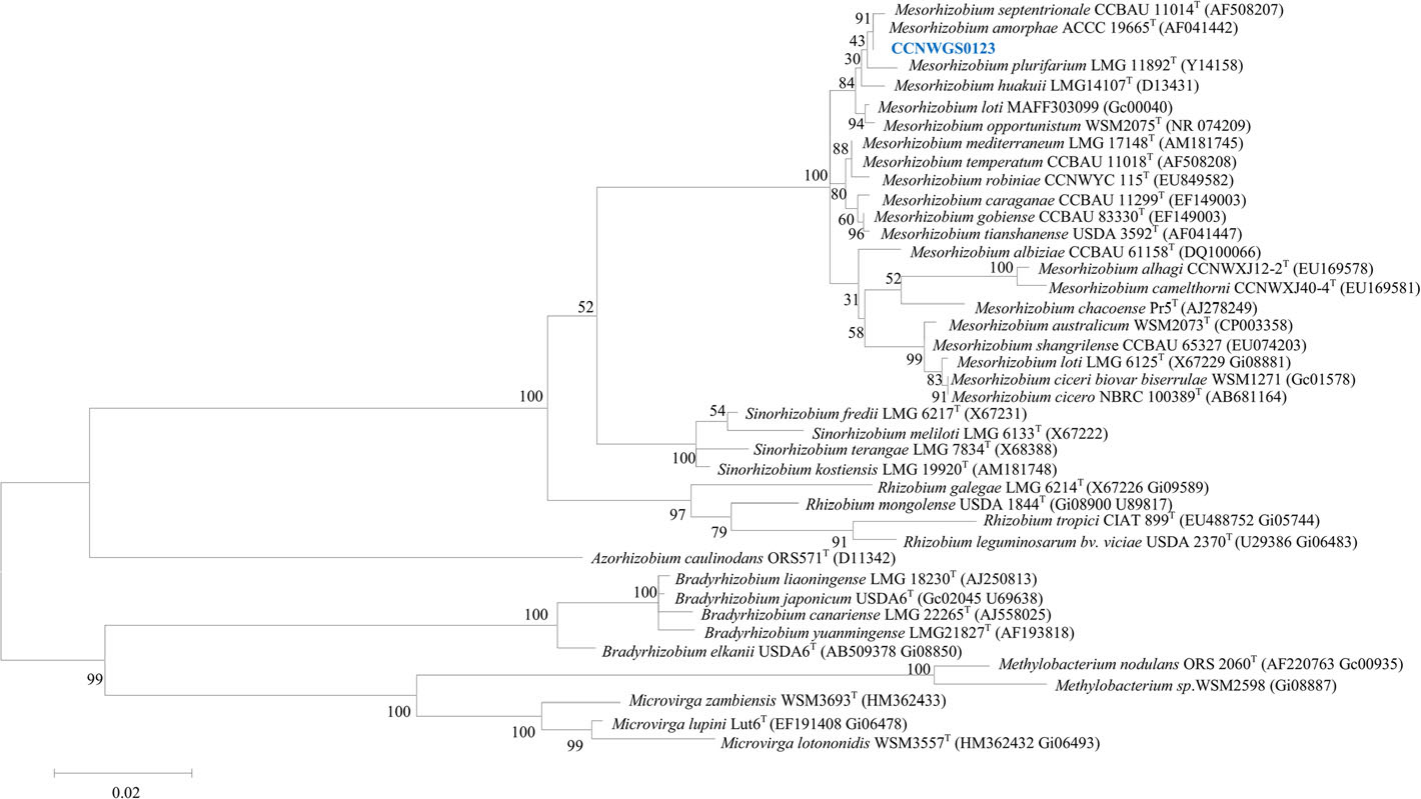
Phylogenetic tree showing the relationships of *Mesorhizobium amorphae* CCNWGS0123 with other root nodule bacteria based on aligned sequences of a 1296 bp internal region of the 16S rRNA gene. All sites were informative and there were no gap-containing sites. Phylogenetic analyses were performed using MEGA, version 6 [15]. The tree was built using the Maximum Likelihood method based on the Tamura-Nei model [16]. Bootstrap analysis [17] with 500 replicates was performed to assess the support of the clusters

*M. amorphae* CCNWGS0123 genome contains two (100% identical) copies of 16S rRNA gene. The phylogenetic neighborhood of *M. amorphae* strain CCNWGS0123 in a 16S rRNA gene sequence-based tree is shown in Fig. 2. Phylogenetic analyses were performed using MEGA version 6 [15]. The evolutionary history was inferred using the Maximum Likelihood method based on the Tamura-Nei model [16]; the percentage of replicate trees to which the associated taxa were clustered in the bootstrap test (500 replicates) are shown next to the branches [17]. *M. amorphae* CCNWGS0123 is phylogenetically closely related to the type strain- *M. amorphae* ACCC 19665^T^, with a 16S rRNA gene sequence identity of 99.93% (1471/1472 bp).

The minimum information about the genome sequence (MIGS) is provided in Table 1.

**Table 1:** Classification and general features of Mesorhizobium amorphae CCNWGS0123

MIGS ID	Property	Term	Evidence code^a^
	Classification	Domain: Bacteria	TAS [39]
		Phylum: *Proteobacteria*	TAS [39, 40]
		Class: *Alphaproteobacteria*	TAS [39, 41, 42]
		Order: *Rhizobiales*	TAS [39, 42, 43]
		Family: *Phyllobacteriaceae*	TAS [39, 42]
		Genus: *Mesorhizobium*	TAS [44, 45]
		Species: *Mesorhizobium amorphae*	TAS [14]
		Stain: CCNWGS0123	
	Gram strain	Negative	NAS
	Cell shape	rod	NAS
	Motility	motile	NAS
	Sporulation	None-spore forming	NAS
	Temperature range	Not reported	
	Optimum temperature	28 °C	NAS
	Carbon source	D xylose, galactose, L-arabinose, D-ribose, rhamnose, mannose, maltose, glucose, saccharose, lactose	TAS
MIGS-6	Habitat	Soil, Host-associated	TAS [13, 39]
MIGS-6.3	Salinity range	Not reported	
MIGS-22	Oxygen requirement	aerobic	NAS
MIGS-15	Biotic relationship	Free living, Symbiont	NAS
MIGS-14	Pathogenicity	Non-pathogen	NAS
MIGS-4	Geographic location	China: Gansu, Huixian	IDA
MIGS-5	Sample collection	2006	IDA
MIGS-4.1	Latitude	33.8 N	IDA
MIGS-4.2	Longitude	106.1E	IDA
MIGS-4.4	Altitude	1049 m	IDA

^a^Evidence codes - IDA Inferred from Direct Assay, TAS Traceable Author Statement (i.e., a direct report exists in the literature), NAS Non-traceable Author Statement (i.e., not directly observed for the living, isolated sample, but based on a generally accepted property for the species, or anecdotal evidence). These evidence codes are from the Gene Ontology project [27]

#### Biochemical profiling

For a detailed biochemical characterization of *M. amorphae* CCNWGS0123, the strain was cultivated for 5 days in 30 ml of Tryptone-Yeast (TY) liquid medium at 28 °C under 200 rpm, and then harvested by centrifugation at 4000 rpm for 5 min. The harvested cells were washed three times by inoculation buffer, resuspended, and diluted in inoculation buffer to reach an optical density of OD_600nm_ = 0.5. The suspension was used for inoculation of Biolog GN2, and GENIII plates (Biolog Inc., USA), and then the plates were incubated for several days at 28 °C. Microtiter plate reader (Bio-Rad, USA) was used for data analysis.

Analyses of the GN2 plates revealed that *M. amorphae* CCNWGS0123 could utilize the following substrates: β-Methyl-D-glucoside, D-galacturonic acid γ- lactone, D-xylose/aldopentose, D-galacturonic acid, I-erythritol, 2-hydroxybenzoic acid, 4-hydroxybenzoic acid, α- cyclodextrin, itaconic acid and D-malic acid. In GENIII plates, the following substrates were utilized: D-maltose, D-trehalose, D-cellobiose, gentiobiose, sucrose, D-turanose, D-raffinose, α-D-lactose, D-melibiose, β-methyl-D-glucoside, D-salicin, N-acetyl-D-glucosamine, N-acetyl-β-D-mannosamine, N-acetyl-D-galactosamine, α-D-glucose, D-mannose, D-fructose, D-galactose, D-fucose, L-fucose, L-rhamnose, D-sorbitol, D-mannitol, D-arabitol, myo-inositol, lycerol, glycyl-L-proline, L-alanine, L-arginine, L-glutamic acid, L-histidine, quinic acid, L-serine, methyl pyruvate, L-lactic acid, D-malic acid and γ-amino-butryric acid.

Compared with the previously described *M. amorphae* type strain- ACCC 19665^T^, *M. amorphae* CCNWGS0123 could not utilize L- phenylalanine, γ- hydroxybutyrate, L-threonine, glycogen, D- glucose histidine, Α-D-lactose, inosine, L-aspartic acid, mucic acid, L-malic acid, bromo-succinic acid in GN2 and GENIII plates.

#### Resilience to abiotic factors and antibiotic resistance

*M. amorphae* CCNWGS0123 could grow on Biolog GenIII plates at an optical density similar to that in positive control at pH 5, pH 6, 1% NaCl, and 1% sodium lactate, and to a lower optical density in lincomycin and nalidixic Acid. This strain could not grow at 4% NaCl or 8% NaCl. Moreover, the growth was inhibited by fusidic acid, D-serine, troleandomycin, rifamycin SV, minocycline, guanidine HCl, Niaproof 4, vancomycin, tetrazolium violet, tetrazolium blue, lithium chloride, potassium tellurite, aztreonam, sodium butyrate and sodium bromate.

#### Symbiotaxonomy

As shown in Additional file 1: Table S1, according to nodulation test, *M. amorphae* CCNWGS0123 is an effective microsymbiont only for woody legumes (*R. pseudoacacia* L. and *A. fruticose*). But the strain could not nodulate with other genera of legume plants, such as *Medicago sativa**.*

## Genome sequencing information

### Genome project history

Because of its ability of heavy metal resistance and establishing symbiosis with *R. pseudoacacia* L., *M. amorphae* CCNWGS0123 was selected for sequencing. Its draft genome sequence was obtained in 2012 using 454 pyrophosphate sequencing technology [10]. To close the gap and correct some mistakes in annotation, the complete genome sequence of *M. amorphae* CCNWGS0123 was obtained in 2015 by using Single Molecule Real-Time (SMRT) technology. Sequencing was performed at Beijing Novogene Bioinformatics Technology Co., Ltd. The final genome assembly of *M. amorphae* CCNWGS0123 is of high quality and completed on five scaffolds (one chromosome and four plasmids) with a sequencing coverage of approximately 134.86 fold. The complete genome sequence of *M. amorphae* CCNWGS0123 was deposited in GenBank (accession numbers CP015318 - CP015322). The project information was summarized in Table 2.

**Table 2:** Project information

MIGS ID	Property	Term
MIGS 31	Finishing quality	High-quality,closed genome
MIGS-28	Libraries used	A 10Kb library
MIGS-29	Sequencing platforms	PacBio RS II
MIGS-31.2	Fold coverage	134.86×
MIGS-30	Assemblers	Celera Assembler CA 8.1
MIGS-32	Gene calling method	GeneMarkS
	Locus Tag	Mea0123
	Genbank ID	Chromosome CP015318; pM0123a CP015319; pM0123b CP015320; pM0123c CP015321; pM0123d CP015322
	Genbank date of Release	July 18,2016
	BIOPROJECT	PRJNA318467
MIGS-13	Source Material Identifier	CCNWGS0123
	Project relevance	Legume plant symbiosis

### Growth conditions and genomic DNA preparation

*M. amorphae* CCNWGS0123 was cultured in TY extract medium and allowed to grow from a single colony at 28 °C in flask agitated under 200 rpm as described previously [18]. Cells were harvested by centrifugation at 5000 rpm, and total DNA was prepared using a TaKaRa MiniBest Bacterial Genomic DNA Extraction Kit Ver. 3.0 (Dalian, China). Thermo Scientific NanoDrop 2000 was used to quantify the DNA in order to ensure that the quality is suitable for sequencing analyses.

### Genome sequencing and assembly

The genome of *M. amorphae* CCNWGS0123 was sequenced using SMRT technology at the Beijing Novogene Bioinformatics Technology Co., Ltd. A 10 kb library was constructed; SMRT Analysis 2.3.0 was used to filter the low-quality reads; and then the filtered reads were assembled to generate scaffold without gaps. The total genome sequence was 7,343,952 bp long, consisting of one chromosome and four plasmids, and with an average coverage of 134.86 fold. The overview of the genome information is shown in Table 3.

**Table 3:** Genome statistics

Attribute	Value	% of total
Genome size (bp)	7,343,952	100
DNA coding (bp)	6,378,582	86.85
DNA G + C (bp)	4,670,753	62.87
DNA scaffolds	4	
Total genes	7378	100
Protein coding genes	7136	96.45
RNA genes	63	0.92
Pseudo genes	179	
Genes in internal clusters	NA	
Genes with function prediction	6726	98.62
Genes assigned to COGs	4758	68.34
Genes with Pfam domains	5805	83.38
Genes with signal peptides	2239	35.72
Genes with transmembrane helices	1585	22.77
CRISPR repeats	4	–

### Genome annotation

Gene prediction was performed by using GeneMarkS (http://topaz.gatech.edu/) with integrated model that combine the GeneMarkS generated (native) and Heuristic model parameters [19]. A whole genome Blast search [20] (E-value is less than 1e-5; minimal alignment length percentage is larger than 40%) was performed against six databases, namely, Kyoto Encyclopedia of Genes and Genomes [21–23], Clusters of Orthologous Groups [24, 25], Non-Redundant Protein Database databases (NR), SwissProt [26] and Gene Ontology [27] and TrEMBL [26]. Transfer RNA (tRNA) genes were predicted using tRNAscan-SE [28]; rRNA genes were predicted using rRNAmmer [29], and small RNA (sRNA) were predicted by BLAST against Rfam [30] database. PHAST [31] was used for prophage prediction (http://phast.wishartlab.com/) and CRISPR Finder [32] was used for CRISPR identification.

## Genome properties

**Fig. 3:**
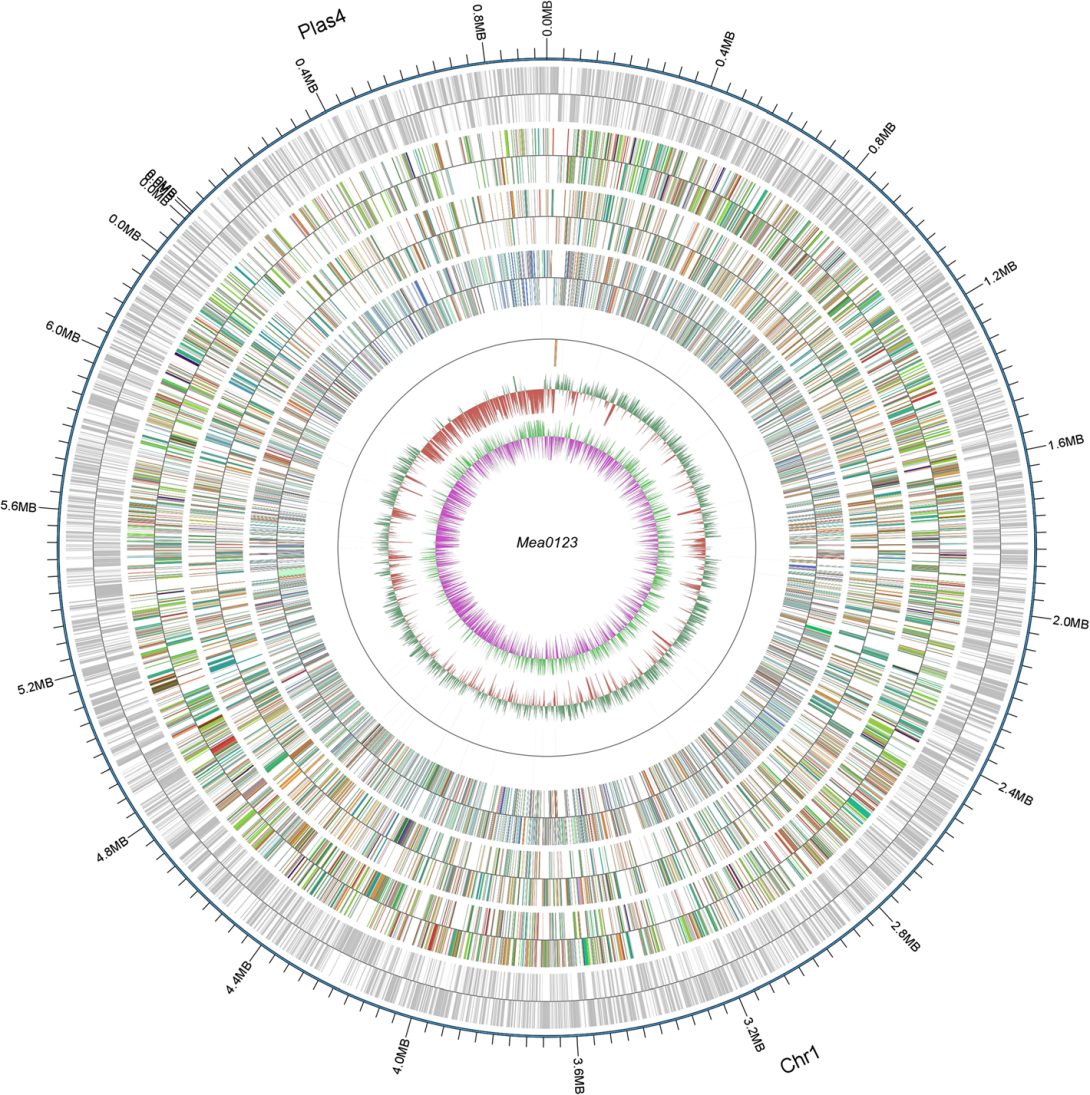
Graphical map of *Mesorhizobium amorphae* CCNWGS0123 genome. From outside to the center: sequence position coordinates, coding gene, COG assignment, KEGG assignment, GO assignment, ncRNA, G + C content and G + C skew

*M. amorphae* CCNWGS0123 genome was consisted of one 6,268,270 bp circular chromosome, one 948,568 bp circular symbiotic plasmid (pM0123d), and three non-circular plasmids (pM0123a-c), whose length ranged from 7607 bp to 102,093 bp (Table 3, Fig. 3). As shown in Table 3, the genome had an average G + C content of 62.87%. The number of predicted genes is 7136. The chromosome contained 53 tRNAs, 4 sRNAs, two copies of 5S, 16S, and 23S rRNA genes. A total of 4758 (66.68%) protein-coding genes were annotated by COG database. The COG assignment of the functional genes is summarized in Table 4. The genome contained highest number of functional genes participating in amino acid transport and metabolism (765), followed by general function prediction only (734). The gene assignments in the six databases are summarized in Table 5. Ten incomplete prophases were identified in chromosome, and two intact prophases were identified in pM0123d. Only four CRISPRs were identified throughout the genome.

**Table 4:** Number of genes associated with the general COG functional categories

Code	Value	% of total	Description
J	190	2.64	Translation, ribosomal structure and biogenesis
A	0	0.00	RNA processing and modification
K	467	6.49	Transcription
L	200	2.78	Replication, recombination and repair
B	4	0.06	Chromatin structure and dynamics
D	26	0.36	Cell cycle control, mitosis and meiosis
V	26	0.36	Defense mechanisms
T	19	0.26	Signal transduction mechanisms
M	253	3.51	Cell wall/membrane biogenesis
N	43	0.60	Cell motility
U	101	1.40	Intracellular trafficking and secretion
O	181	2.51	Posttranslational modification, protein turnover, chaperones
C	328	4.56	Energy production and conversion
G	492	6.83	Carbohydrate transport and metabolism
E	765	10.63	Amino acid transport and metabolism
F	66	0.92	Nucleotide transport and metabolism
H	189	2.63	Coenzyme transport and metabolism
I	233	3.24	Lipid transport and metabolism
P	283	3.93	Inorganic ion transport and metabolism
Q	197	2.74	Secondary metabolites biosynthesis, transport and catabolism
R	734	10.20	General function prediction only
S	485	6.74	Function unknown
–	1690	23.48	Not in COGs

**Table 5:** Function annotation assignment from different databases

Database	Assigned Number	Percent (%)
COG	4758	66.68
GO	4163	58.34
KEGG	3700	51.85
NR	6726	94.25
Swissprot	2268	31.78
TrEMBL	6096	85.43
Annotated	6962	97.56
Total	7136	100

## Extended insights from the genome sequence

### Genomic comparison between *M. amorphae* CCNWGS0123 and other *Mesorhizobium* species

**Fig. 4:**
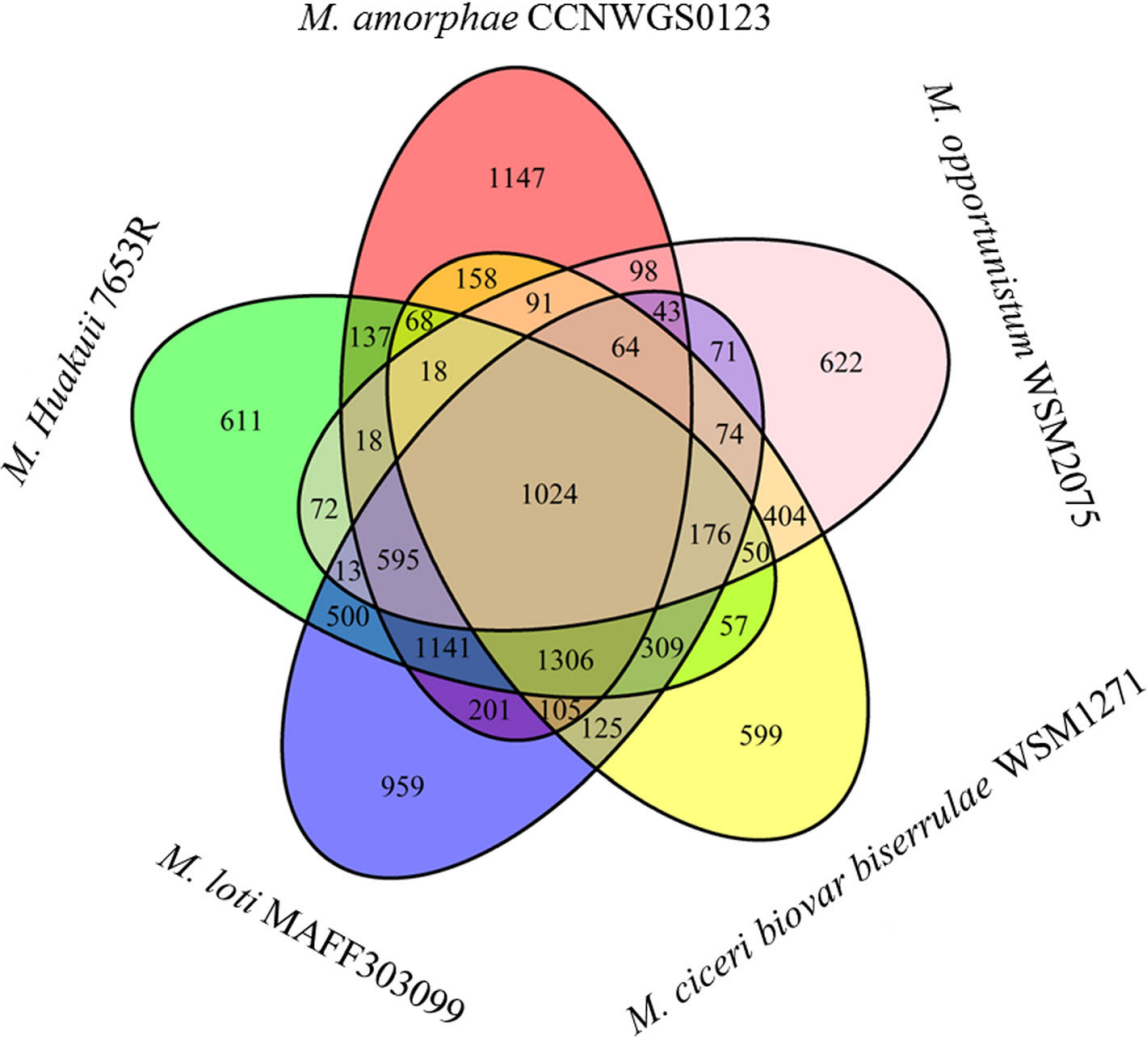
Core and accessory genome analysis of five *Mesorhizobium* strains

The genome of *M. amorphae* CCNWGS0123 was compared with those of four *Mesorhizobium* strains, including *M. huakuii* 7653R, *M. loti* MAFF303099, *M. ciceri* WSM1271 and *M. opportunistum* WSM2075. The general features of the five *Mesorhizobium* genomes were summarized in Table 6. Totally, 6918 orthologous groups of genes were identified in the five *Mesorhizobium* strains. Among these groups, 1024 groups were conserved among the five genomes, and these orthologous groups were termed as the core genome of the five *Mesorhizobium* genomes (Fig. 4). Additionally, 2159 orthologous groups were present in four of the five genomes; 1912 orthologous groups were found in three genomes; and the remaining 1833 orthologous groups are present in two genomes. *M. amorphae* CCNWGS0123 had 1147 strain specific genes, occupied 16.07% of the total coding genes.

**Table 6:** General Information of five *Mesorhizobium* genome

	CCNWGS0123	7653R	MAFF303099	WSM1271	WSM2075
length	7,343,952	6,881,676	7,569,297	6,690,028	6,884,444
G + C%	62.8	62.86	62.51	62.56	62.87
gene	7378	6661	7298	6532	6576
CDS	7136	6235	7076	6264	6418
RNA	63	55	60	62	61

### Metabolism pathway

**Fig. 5:**
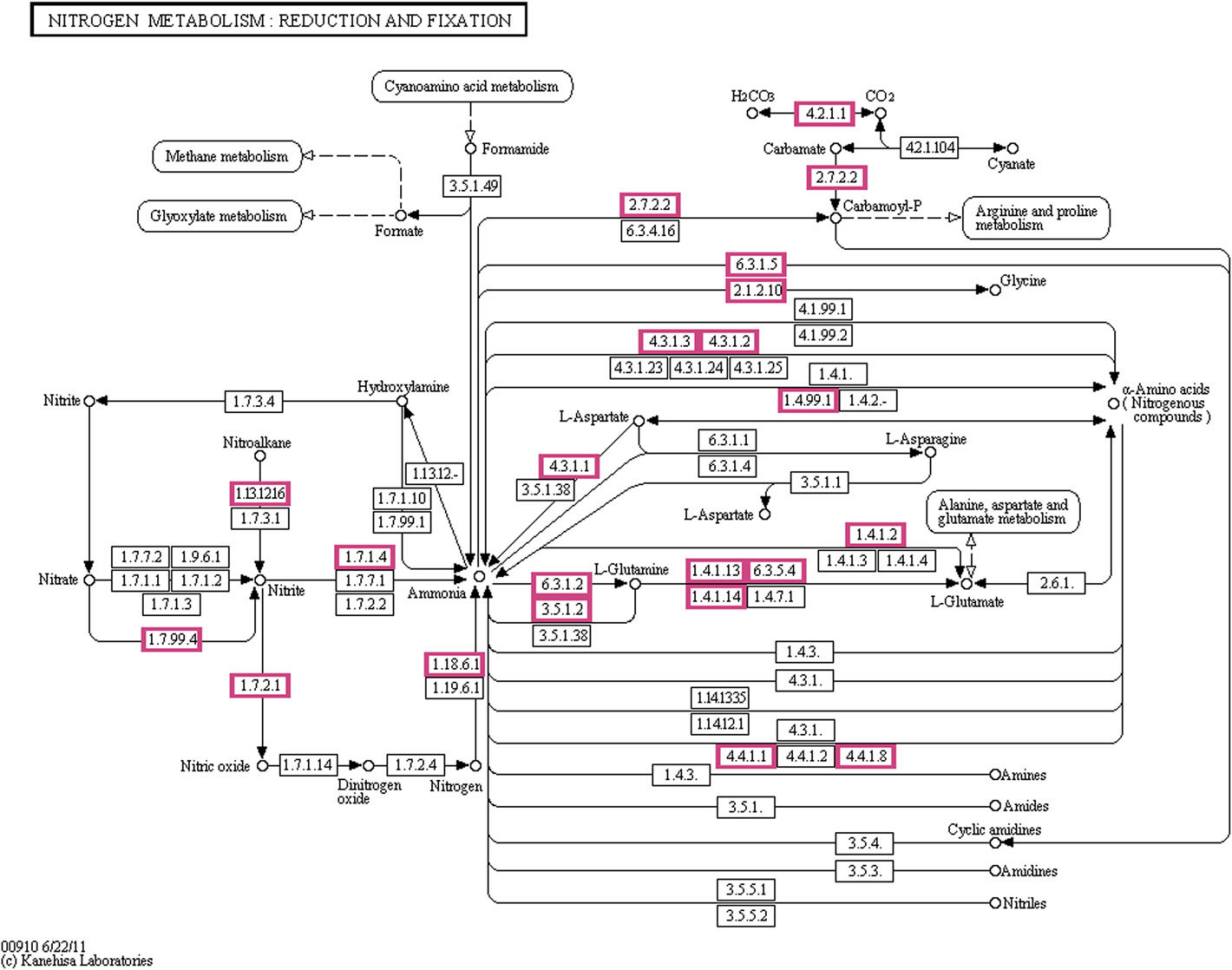
The pathway of synthesis and degradation of nitrogen

A total of 3700 genes could find their corresponding genes in the KEGG database; these genes participate in 132 KEGG metabolism pathways (Additional file 2: Table S2), including amino acid metabolism, carbohydrate metabolism, and nucleotide metabolism pathways. A specific metabolism pathway, namely, Nitrogen metabolism was observed in *M. amorphae* CCNWGS0123 (Fig. 5), 48 genes participate in nitrogen biosynthesis and degradation (Additional file 3: Table S3). Three genes, *nif*K, *nif*D and *nif*H participate in biosynthesis of the key enzyme- nitrogenase.

### Nitrogen fixation genes

Nitrogen fixation related genes homologous to N_2_ fixation genes in *Klebsiella pneumoniae* [33, 34] are referred to as *nif* genes; the other genes which are also essential in symbiotic N_2_ fixation but sharing no homology to *K. pneumoniae* are called *fix* genes [35]. A total of 29 *nif*/*fix* genes were found in *M. amorphae* CCNWGS0123 genome (Additional file 4: Table S4), and most of these genes display a relatively high similarity with those of other *Mesorhizobium* species based on amino acid sequences, except for NifV (< 35%).

### Nodulation genes

Rhizobia could establish symbiotic interactions with many legume species, and convert atmospheric N_2_ into ammonium. In rhizobial strains, two cluster genes, namely, nodulation and nitrogen fixation genes, play crucial roles in these processes [2, 36]. Nodulation factors (NFs), as key signals in rhizobia, are encoded by three groups of nodulation genes. The first group contained common *nod* genes, whose products are required in the backbone of NF structrures (*nodABC*); these genes are present in nearly all of rhizobia strains. The second group included the host-specific *nod* genes participating in species-specific modifications of the NF core (*nodEF*, *nodG*, *nodH*, *nodPQ* and *nodRL*). The third group included the regulatory genes (*nodD*, *nolR* and *nodVW*) [37, 38].

As shown in Additional file 5: Table S5, *M. amorphae* CCNWGS0123 genome contained 12 nodulation genes. Compared with the other four *Mesorhizobium* strains, *M. amorphae* CCNWGS0123 contained the lowest number of nodulation genes. Moreover, most of the proteins encoded by these genes displayed low sequence similarities with the corresponding proteins in other *Mesorhizobium* strains based on amino acid sequences, with exceptions of NodF (> 95%) and NodN (> 97%).

### Genes related to heavy metal resistance

*M. amorphae* CCNWGS0123 was isolated from *R. pseudoacacia* L. nodules who grown in lead-zinc mine tailing site, the strain could help its host plant to survive in copper-, zinc-, and chromium-contaminated environments [9, 10]. The strain possesses multiple heavy metal tolerance and equilibrium ability [9]. Compared with other *Mesorhizobium* strains, *M. amorphae* CCNWGS0123 contained more genes participating in heavy metal resistance and transport. As shown in Additional file 6: Table S6, a total of 46 genes participating in heavy mental (Ag, As, Cd, Co, Cu, Hg, Mo or Zn) resistance and transport were identified in *M. amorphae* CCNWGS0123 genome. Genes participating in heavy mental resistance and transport were also identified in other *Mesorhizobium* genomes, 32 genes were identified in *M. huakuii* 7653R genome, 35 genes were identified in *M. loti* MAFF303099 genome, 28 genes were identified in *M. ciceri* WSM1271 genome and 26 genes were identified in *M. opportunistum* WSM2075 genome.

Compared with the other four strains, *M. amorphae* CCNWGS0123 contained 10 specific genes involved in heavy mental As (*mea0123GM001797*, *mea0123GM002757*, *mea0123GM004652* and *mea0123GM006759*), Cd/Zn/Co (*mea0123GM001790* and *mea0123GM004338*), Cu (*mea0123GM001765*, *mea0123GM006395*, *mea0123GM006849*) and Cu/Ag (*mea0123GM001789*) resistance and transport and one CadZ encoding gene (*mea0123GM000975*). These genes may play important roles in helping survival in heavy mental-contaminated soil.

## Conclusions

The previous study presents the complete genome sequence of *M. amorphae* CCNWGS0123 which was isolated from *R. pseudoacacia* L. grown in lead-zinc mine tailing site. A total of 46 genes involved in heavy metal tolerance were identified in the whole genome sequence. As predicted by Wang [14], *M. amorphae* strains harbor one 0.9 Mb symbiotic plasmid. *M. amorphae* CCNWGS0123 genome contains a circular symbiotic plasmid with 0.95 Mb. Symbiosis related genes (nodulation and nitrogen fixation genes) were found in the symbiotic plasmid (pM0123d). Compared with other *Mesorhizobium* stains, *M. amorphae* CCNWGS0123 contained different number and genetic constitution of symbiosis genes. The complete genome sequence of *M. amorphae* CCNWGS0123 will provide some bases in studying the heavy metal tolerance mechanism and signal regulation during symbiosis process.

## Additional files

Additional file 1: **Table S1.** Compatibility of *M. amorphae* CCNWGS0123 with different wild and cultivated legume species. 13 genera and 14 species legume plants were grown in perlite and vermiculite (1:2) mixture substance, nodule number was calculated 30 days after inoculation of *M. amorphae* CCNWGS0123. (DOCX 19 kb)

Additional file 2: **Table S2.** KO numbers of M. amorphae CCNWGS0123. (DOCX 17 kb)

Additional file 3: **Table S3.** Genes participating in nitrogen synthesis and degradation. (DOCX 18 kb)

Additional file 4: **Table S4.** Nitrogen fixation protein similarities between M. amorphae CCNWGS0123 and other four *Mesorhizobium* strains. (DOCX 21 kb)

Additional file 5: **Table S5.** Nodulation protein similarities between *M. amorphae* CCNWGS0123 and other four *Mesorhizobium* strains. (DOCX 20 kb)

Additional file 6: **Table S6.** Genes involved in heavy metal resistant and homeostasis throughout the whole genome. (XLSX 13 kb)

## Abbreviations

COG: Clusters of Orthologous Groups
GO: Gene Ontology
KEGG: Kyoto Encyclopedia of Genes and Genomes
NR: Non-Redundant Protein Database databases
SEM: scanning electron microscope
