# Supplementary material for: Complete genome sequence of the Robinia pseudoacacia L. symbiont Mesorhizobium amorphae CCNWGS0123
Source: Stand Genomic Sci. 2018 Sep 18;13:18. doi: 10.1186/s40793-018-0321-3 (PMC6145117; doi:10.1186/s40793-018-0321-3)
Supplement: Supplementary file 1 — Table S1. Compatibility of M. amorphae CCNWGS0123 with different wild and cultivated legume species. 13 genera and 14 species legume plants were grown in perlite and vermiculite (1:2) mixture substance, nodule number was calculated 30 days after inoculation of M. amorphae CCNWGS0123. (DOCX 19 kb) [file 40793_2018_321_MOESM1_ESM.docx]

Table S1 Compatibility of *Mesorhizobium amorphae* CCNWGS0123 with different wild and cultivated legume species

| Species | Genera | Family | Habit/Growth type | Nod | Fix |
| --- | --- | --- | --- | --- | --- |
| *Amorpha fruticosa.* | [*Amorpha*](https://en.wikipedia.org/wiki/Amorpha)*.* | *Fabaceae* | Perennial shrub | + | + |
| *Robinia pseudoacacia.* | [*Robinia*](https://en.wikipedia.org/wiki/Robinia)*.* | *Fabaceae* | Perennial arbor | + | + |
| *Medicago sativa.* | [*Medicago*](https://en.wikipedia.org/wiki/Medicago)*.* | *Fabaceae* | Perennial herbaceous | - | - |
| *Melilotus albus.* | [*Melilotus*](https://en.wikipedia.org/wiki/Melilotus)*.* | *Fabaceae* | Biennial herbaceous | - | - |
| *Securigera varia.* | [*Securigera*](https://en.wikipedia.org/wiki/Securigera)*.* | *Fabaceae* | Perennial herbaceous | - | - |
| *Astragalus adsurgens Pall.* | *Astragalus Linn.* | *Fabaceae* | Biennial herbaceous | - | - |
| *Licia villosa Roth.* | *Vicia Linn.* | *Fabaceae* | Annual herbaceous | - | - |
| *Kummerowia striata* | *Kummerowia Schindl.* | *Fabaceae* | Annual herbaceous | - | - |
| *Trifolium repens L.* | *Trifolium Linn.* | *Fabaceae* | Perennial herbaceous | - | - |
| *Trifolium pretense.* | *Trifolium Linn.* | *Fabaceae* | Perennial herbaceous | - | - |
| *Lespedeza bicolor Turcz.* | *Lespedeza Michx.* | *Fabaceae* | Perennial shrub | - | - |
| *Albizia julibrissin Durazz.* | *Albizia Durazz* | *Fabaceae* | Perennial arbor | - | - |
| *Ammopiptanthus mongolicu.s* | *Ammopiptanthus Cheng f.* | *Fabaceae* | Perennial shrub | - | - |
| *Vigna unguiculata.* | *Vigna Savi* | *Fabaceae* | Annual  herbaceous | - | - |

Legume plants were grown in perlite and vermiculite (1: 2) mixture substance, nodule number was calculated 30 days after inoculation of CCNWGS0123.
