# Supplementary material for: Complete genome sequence of the Robinia pseudoacacia L. symbiont Mesorhizobium amorphae CCNWGS0123
Source: Stand Genomic Sci. 2018 Sep 18;13:18. doi: 10.1186/s40793-018-0321-3 (PMC6145117; doi:10.1186/s40793-018-0321-3)
Supplement: Supplementary file 2 — Table S2. KO numbers of M. amorphae CCNWGS0123. (DOCX 17 kb) [file 40793_2018_321_MOESM2_ESM.docx]

Table S2 KO numbers of *M. amorphae* CCNWGS0123

| KO numbers of *M. amorphae* CCNWGS0123 | | | | | | | |
| --- | --- | --- | --- | --- | --- | --- | --- |
| ko00010 | ko00020 | ko00030 | ko00040 | ko00051 | ko00052 | ko00053 | ko00061 |
| ko00071 | ko00120 | ko00130 | ko00140 | ko00190 | ko00194 | ko00195 | ko00230 |
| ko00232 | ko00240 | ko00250 | ko00260 | ko00270 | ko00280 | ko00281 | ko00290 |
| ko00300 | ko00310 | ko00311 | ko00330 | ko00340 | ko00350 | ko00351 | ko00360 |
| ko00361 | ko00362 | ko00363 | ko00364 | ko00380 | ko00400 | ko00410 | ko00430 |
| ko00440 | ko00450 | ko00471 | ko00473 | ko00480 | ko00500 | ko00520 | ko00523 |
| ko00540 | ko00550 | ko00561 | ko00562 | ko00564 | ko00590 | ko00600 | ko00620 |
| ko00621 | ko00625 | ko00627 | ko00630 | ko00633 | ko00640 | ko00650 | ko00660 |
| ko00670 | ko00680 | ko00710 | ko00720 | ko00730 | ko00740 | ko00750 | ko00760 |
| ko00770 | ko00780 | ko00785 | ko00790 | ko00791 | ko00860 | ko00900 | ko00903 |
| ko00910 | ko00920 | ko00930 | ko00941 | ko00960 | ko00970 | ko01001 | ko01002 |
| ko01003 | ko01004 | ko01005 | ko01006 | ko01007 | ko01040 | ko01053 | ko02000 |
| ko02020 | ko02022 | ko02030 | ko02035 | ko02040 | ko02042 | ko02044 | ko03000 |
| ko03008 | ko03009 | ko03010 | ko03012 | ko03013 | ko03018 | ko03021 | ko03030 |
| ko03032 | ko03036 | ko03060 | ko03070 | ko03110 | ko03400 | ko03410 | ko03420 |
| ko03430 | ko03440 | ko03450 | ko04040 | ko04112 | ko04121 | ko04122 | ko04142 |
| ko04146 | ko04210 | ko04978 | ko05322 |  |  |  |  |
