# Supplementary material for: Complete genome sequence of the Robinia pseudoacacia L. symbiont Mesorhizobium amorphae CCNWGS0123
Source: Stand Genomic Sci. 2018 Sep 18;13:18. doi: 10.1186/s40793-018-0321-3 (PMC6145117; doi:10.1186/s40793-018-0321-3)
Supplement: Supplementary file 3 — Table S3. Genes participating in nitrogen synthesis and degradation. (DOCX 18 kb) [file 40793_2018_321_MOESM3_ESM.docx]

Table S3 Genes participating in nitrogen synthesis and degradation

| Gene ID | Annotation |
| --- | --- |
| *mea0123GM000008* | glutamine synthetase |
| *mea0123GM000232* | glycine cleavage system protein T |
| *mea0123GM000450* | FAD dependent oxidoreductase |
| *mea0123GM000461* | D-amino acid dehydrogenase small subunit |
| *mea0123GM000535* | NAD-glutamate dehydrogenas |
| *mea0123GM000567* | carbonate dehydratase |
| *mea0123GM000819* | Cys/Met metabolism pyridoxal-phosphate-dependent protein |
| *mea0123GM000820* | D-amino acid dehydrogenase |
| *mea0123GM001187* | glutamine synthetase |
| *mea0123GM001334* | asparagine synthase |
| *mea0123GM001661* | D-amino acid dehydrogenase |
| *mea0123GM001706* | carbamate kinase |
| *mea0123GM001869* | glutamate--putrescine ligase |
| *mea0123GM002105* | histidine ammonia-lyase |
| *mea0123GM002227* | hypothetical protein |
| *mea0123GM002267* | glycine cleavage T protein (aminomethyl transferase) |
| *mea0123GM002272* | glutamine synthetase III |
| *mea0123GM002282* | glycine cleavage T protein (aminomethyl transferase) |
| *mea0123GM002831* | Nitrate reductase |
| *mea0123GM003241* | cystathionine beta-lyase |
| *mea0123GM003393* | FAD dependent oxidoreductase |
| *mea0123GM003438* | histidine ammonia-lyase |
| *mea0123GM003476* | nitrite reductase (NAD(P)H), large subunit |
| *mea0123GM003477* | nitrite reductase (NAD(P)H), small subunit |
| *mea0123GM003478* | molybdopterin oxidoreductase |
| *mea0123GM004069* | glutamine synthetase, type I |
| *mea0123GM004070* | glutamate--ammonia ligase |
| *mea0123GM004172* | glutaminase |
| *mea0123GM004243* | NAD synthetase |
| *mea0123GM004289* | glycine cleavage system aminomethyltransferase T |
| *mea0123GM004755* | cystathionine gamma-lyase |
| *mea0123GM004811* | dihydropyrimidine dehydrogenase subunit A |
| *mea0123GM004865* | histidine ammonia-lyase |
| *mea0123GM005085* | nitrite reductase |
| *mea0123GM005783* | dihydropyrimidine dehydrogenase subunit A |
| *mea0123GM005784* | glutamate synthase, large subunit |
| *mea0123GM005829* | glutamine synthetase |
| *mea0123GM006081* | glutamate--putrescine ligase |
| *mea0123GM006585* | 3-methylaspartate ammonia-lyase |
| *mea0123GM006606* | 3-methylaspartate ammonia-lyase |
| *mea0123GM006679* | nitrogenase molybdenum-iron protein beta chain, nifK |
| *mea0123GM006680* | nitrogenase molybdenum-iron protein alpha chain, nifD |
| *mea0123GM006681* | Nitrogenase (molybdenum-iron) reductase and maturation protein NifH |
| *mea0123GM006804* | aspartate ammonia-lyase |
| *mea0123GM006893* | a-type carbonic anhydrase |
| *mea0123GM006897* | asparagine synthase |
| *mea0123GM007054* | conserved hypothetical protein |
| *mea0123GM007072* | conserved hypothetical protein |
