# Supplementary material for: Complete genome sequence of the Robinia pseudoacacia L. symbiont Mesorhizobium amorphae CCNWGS0123
Source: Stand Genomic Sci. 2018 Sep 18;13:18. doi: 10.1186/s40793-018-0321-3 (PMC6145117; doi:10.1186/s40793-018-0321-3)
Supplement: Supplementary file 4 — Table S4. Nitrogen fixation protein similarities between M. amorphae CCNWGS0123 and other four Mesorhizobium strains. (DOCX 21 kb) [file 40793_2018_321_MOESM4_ESM.docx]

Table S4 Nitrogen Fixation protein identities between *M. amorphae* CCNWGS0123 and other four *Mesorhizobium* strains

|  | *M.amorpahe* CCNWGS0123 | *M.huakui* 7653R | *M.ciceri* WSM1271 | *M.loti* MAFF303099 | *M.opportunistum* WSM2705 |
| --- | --- | --- | --- | --- | --- |
| FdxN | Mea0123GM006752 | MCHK_8161(80%) | Mesci_5841(96%) | msl8750(88%) | Mesop_6415(86%) |
| FixA | Mea0123GM006746 | MCHK_8217(89%) | Mesci_5836(87%) | mll5862(86%) | Mesop_6410(87%) |
| FixB | Mea0123GM006747 | MCHK_8218(92%) | Mesci_5837(88%) | mll5861(89%) | Mesop_6411(88%) |
| FixC | Mea0123GM006748 | MCHK_8219(90%) | Mesci_5838(89%) | mll5860(87%) | Mesop_6412(89%) |
| FixG | Mea0123GM005096 | MCHK_0354(87%) | Mesci_6045(88%) | mll6626(88%) | Mesop_6030(86%) |
| FixG |  |  | Mesci_5506(84%) | mlr6415(85%) | Mesop_6087(84%) |
| FixH | Mea0123GM006267 | MCHK_0353(77%) | Mesci_5505(76%) | mll6625(76%) | Mesop_6086(76%) |
| FixH |  |  | Mesci_6044(78%) | mlr6416(74%) | Mesop_6031(79%) |
| FixI | Mea0123GM005098 | MCHK_0352(81%) | Mesci_6043(85%) | mll6624(86%) | Mesop_6032(85%) |
| FixI |  |  | Mesci_5504(84%) | mlr6417(84%) | Mesop_6085(84%) |
| FixJ | Mea0123GM005105 | MCHK_3913(82%) | Mesci_6462(87%) | mll6606(87%) | Mesop_6045(86%) |
| FixJ | Mea0123GM001732 |  |  |  |  |
| FixK |  | MCHK_3897 | Mesci_6459 | mll6578 | Mesop_6050 |
| FixK |  | MCHK_3951 |  |  |  |
| FixL |  | MCHK_3912 | Mesci_6463 | mll6607 | Mesop_6044 |
| FixL |  | MCHK_8231 |  |  |  |
| FixN | Mea0123GM006272 | MCHK_0358(95%) | Mesci_6049(93%) | mll6630(95%) | Mesop_6026(94%) |
| FixN |  |  | Mesci_5510(90%) | mlr6411(92%) | Mesop_6091(90%) |
| FixO | Mea0123GM006271 | MCHK_0357(92%) | Mesci_5509(93%) | mll6629(92%) | Mesop_6027(94%) |
| FixO |  |  | Mesci_6048(95%) | mlr6412(92%) | Mesop_6090(93%) |
| FixP | Mea0123GM006269 | MCHK_0355(87%) | Mesci_5507(84%) | mll6628(87%) | Mesop_6029(90%) |
| FixP |  |  | Mesci_6046(89%) | mlr6414(87%) | Mesop_6088(84%) |
|  | *M.amorpahe* CCNWGS0123 | *M.huakui* 7653R | *M.ciceri* WSM1271 | *M.loti* MAFF303099 | *M.opportunistum* WSM2705 |
| FixQ | Mea0123GM006270 | MCHK_0356(85%) | Mesci_6047(94%) | msl6627(83%) | Mesop_6028(88%) |
| FixQ |  |  | Mesci_5508(83%) | msr6413(79%) | Mesop_6089(83%) |
| FixS | Mea0123GM005099 | MCHK_0351(100%) |  | msl6623(80%) |  |
| FixS |  |  |  | msr6418(80%) |  |
| FixT |  | MCHK_8164 |  |  |  |
| FixU | Mea0123GM006754 |  | Mesci_5843(77%) | msl5852(84%) | Mesop_6417(77%) |
| FixX | Mea0123GM006749 | MCHK_8220(84%) | Mesci_5839(87%) | msl5859(87%) | Mesop_6413(87%) |
| NifA | Mea0123GM006750 | MCHK_8214(56%) | Mesci_5851(47%) | mll5857(76%) | Mesop_6425(47%) |
| NifA |  |  |  | mll5837(52%) |  |
| NifB | Mea0123GM006751 | MCHK_8226(84%) | Mesci_5840(81%) | mll5855(83%) | Mesop_6414(81%) |
| NifD | Mea0123GM006680 | MCHK_8175(92%) | Mesci_5816(91%) | mlr5906(91%) | Mesop_6392(91%) |
| NifE | Mea0123GM006678 | MCHK_8172(89%) | Mesci_5814(85%) | mlr5908(88%) | Mesop_6390(85%) |
| NifH | Mea0123GM006681 | MCHK_8176(96%) | Mesci_5817(96%) | MLR5905(95%) | Mesop_6393(96%) |
| NifK | Mea0123GM006679 | MCHK_8174(95%) | Mesci_5815(92%) | mlr5907(93%) | Mesop_6391(92%) |
| NifN | Mea0123GM006677 | MCHK_8171(83%) | Mesci_5813(81%) | mlr5909(85%) | Mesop_6389(81%) |
| NifQ | Mea0123GM006692 | MCHK_8188(74%) | Mesci_5823(70%) | mlr5871(74%) | Mesop_6398(70%) |
| NifS | Mea0123GM006743 | MCHK_1835(35%) | Mesci_5833(78%) | mll5865(76%) | Mesop_6407(78%) |
| NifS |  |  | Mesci_4390(36%) | mlr0015(35%) | Mesop_4834(34%) |
| NifU |  | MCHK_2657 | Mesci_4207 | mll0920 | Mesop_4044 |
| NifV | Mea0123GM006744 | MCHK_1235(31%) | Mesci_4792(31%) | mlr7805(32%) | Mesop_5287(31%) |
| NifW | Mea0123GM006745 |  | Mesci_5835(79%) | mll5864(76%) | Mesop_6409(79%) |
| NifX | Mea0123GM006676 | MCHK_8169(pseudo) | Mesci_5812(79%) | mlr5911(90%) | Mesop_6388(79%) |
| NifZ | Mea0123GM006753 | MCHK_8163(78%) | Mesci_5842(76%) | mll5854(80%) | Mesop_6416(76%) |
