# Supplementary material for: Complete genome sequence of the Robinia pseudoacacia L. symbiont Mesorhizobium amorphae CCNWGS0123
Source: Stand Genomic Sci. 2018 Sep 18;13:18. doi: 10.1186/s40793-018-0321-3 (PMC6145117; doi:10.1186/s40793-018-0321-3)
Supplement: Supplementary file 5 — Table S5. Nodulation protein similarities between M. amorphae CCNWGS0123 and other four Mesorhizobium strains. (DOCX 20 kb) [file 40793_2018_321_MOESM5_ESM.docx]

Table S5 Nodulation protein identities between *M. amorphae* CCNWGS0123 and other four *Mesorhizobium* strains

|  | *M.amorphae*  CCNWGS0123 | *M.huakuii*  7653R | *M.ciceri*  WSM1271 | *M.loti*  MAFF303099 | *M.opportunistum*  WSM2075 |
| --- | --- | --- | --- | --- | --- |
| NodA | Mea0123GM006773 | MCHK_8321 (62%) | Mesci_5862 (62%) | mlr8755 (69%) | Mesop_6436 (62%) |
| NodB | Mea0123GM006772 | MCHK_8285 (67%) | Mesci_5863 (64%) | mlr6175 (71%) | Mesop_6437 (64%) |
| NodC | Mea0123GM006771 | MCHK_8284 (68%) | Mesci_5864 (73%) | mlr6163 (75%) | Mesop_6438 (73%) ) |
| NodD1 | Mea0123GM006778 | - | Mesci_5855 (66%) | mll6179 (73%) | Mesop_6429 (66%) |
| NodD2 | - | MCHK_8286 (68%) | Mesci_5861 (66%) | - | Mesop_6435 (66%) |
| NodD3 | - | - | - | mlr6182 (70%) | - |
| NodE | - | MCHK_6007 | Mesci_0524 | mlr4953 | Mesop_0539 |
| NodE | - | MCHK_8324 | Mesci_5858 | mlr5822 | Mesop_6432 |
| NodF | Mea0123GM001102 | MCHK_6006 (96%) | Mesci_0525 (95%) | mlr4951 (98%) | Mesop_0540 (95%) |
| NodF | - | MCHK_8323 (55%) | Mesci_5857 (52%) | mlr5821 (53%) | Mesop_6431 (52%) |
| NodG | - | MCHK_1282 | Mesci_4951 | mlr7850 | Mesop_5244 |
| NodG | - | MCHK_8325 | Mesci_5859 | - | Mesop_6433 |
| NodH | Mea0123GM006760 | MCHK_8281 (63%) | - | - | - |
| NodI | Mea0123GM006768 | MCHK_8283 (80%) | Mesci_5865 (84%) | mlr6164 (84%) | Mesop_6439 (84%) ) |
| NodJ | Mea0123GM006767 | MCHK_8282 (76%) | Mesci_5866 (77%) | mlr6166 (78%) | Mesop_6440 (77%) |
| NodL | - | MCHK_4332 | Mesci_2106 | mll2768 | Mesop_2223 |
| NodM | - | MCHK_7093 | Mesci_4136 | mlr6774 | Mesop_4114 |
| NodN | Mea0123GM005838 | MCHK_4592 (98%) | Mesci_1825 (98%) | mlr3097 (99%) | Mesop_1868 (97%) |
| NodN | Mea0123GM004429 | - | - | - | - |
|  | *M.amorphae*  CCNWGS0123 | *M.huakuii*  7653R | *M.ciceri*  WSM1271 | *M.loti*  MAFF303099 | *M.opportunistum*  WSM2075 |
| NodP | - | MCHK_1040 | Mesci_4953 | mlr7575 | Mesop_5464 |
| NodQ | - | MCHK_1041 | Mesci_4952 | mlr7576 | Mesop_5463 |
| NodS | Mea0123GM006770 | - | - | mlr6161 (72%) | - |
| NodT | - | MCHK_2751 | Mesci_3479 | mll1107 | Mesop_3967 |
| NodU | Mea0123GM006769 | - | Mesci_5894 (70%) | - | Mesop_6463 (70%) |
| NodW | - | - | - | - | Mesop_5572 |
| NodZ | - | - | - | mlr5848 | - |
| NoeJ | - | - | - | mlr5802 | - |
| NoeK | - | - | - | mlr5801 | - |
| NoeL | - | - | - | mlr5849 | - |
| NolA | - | MCHK_0037 | Mesci_0562 | mlr5623 | Mesop_6654 |
| NolK | - | - | - | mlr8749 | - |
| NolL | - | - | - | mlr8757 | - |
| NolO | - | - | Mesci_5868 | mlr6171 | Mesop_6442 |
| NolR | - | MCHK_4356 | Mesci_2083 | mlr2794 | Mesop_2199 |
